# Adiponectin‐expressing Treg‐containing T cell fraction inhibits tumor growth in orthotopically implanted triple‐negative breast cancer

**DOI:** 10.1111/1759-7714.15102

**Published:** 2023-09-06

**Authors:** Wakana Chikaishi, Toshiya Higashi, Hirokatsu Hayashi, Yuki Hanamatsu, Manabu Futamura, Nobuhisa Matsuhashi, Chiemi Saigo, Tamotsu Takeuchi

**Affiliations:** ^1^ Department of Gastroenterological Surgery and Pediatric Surgery Gifu University Graduate School of Medicine Gifu Japan; ^2^ Department of Pathology and Translational Research Gifu University Graduate School of Medicine Gifu Japan; ^3^ Department of Breast Surgery Gifu University Hospital Gifu Japan; ^4^ The United Graduate School of Drug Discovery and Medical Information Sciences Gifu University Gifu Japan; ^5^ Center for One Medicine Innovative Translational Research; COMIT Gifu University Gifu Japan

**Keywords:** adiponectin, adoptive cell therapy, A‐TregTF, cell‐in‐cell, triple‐negative breast cancer

## Abstract

**Background:**

In our previous study, we identified a population of adiponectin expressing regulatory T cells (Tregs) residing within thymic nurse cell complexes, which were capable of inhibiting the development of breast cancer in vitro. Triple‐negative breast cancer (TNBC) with no proper treatment at present is characterized by the absence of estrogen receptor, progesterone receptor, and human epidermal growth factor receptor‐2. In this study, we aimed to investigate the potential of a cultured T cell fraction comprising adiponectin‐expressing Tregs, referred to as A‐TregTF (adiponectin‐expressing Treg‐containing T cell fraction), in inhibiting the progression of TNBC in vivo.

**Methods:**

The efficacy of a spontaneously expanding T cell fraction comprising adiponectin‐expressing Treg in inhibiting tumor growth was analyzed in a murine orthotopic 4 T1‐Luc TNBC model.

**Results:**

The treatment with T cell fraction containing adiponectin‐expressing Tregs significantly inhibited the growth and metastasis of orthotopically transplanted 4 T1‐Luc tumor cells. Histopathological examination further revealed that the adiponectin‐expressing Tregs infiltrated the tumor tissue via a cell‐in‐cell mechanism and were found to be specifically localized around the necrotic areas.

**Conclusions:**

Based on our findings, the T cell fraction comprising adiponectin‐expressing Tregs, represents a potential candidate for adoptive cell therapy against TNBC.

## Graphical abstract

The treatment with T cell fraction containing adiponectin‐expressing Tregs, designated A‐TregTF significantly inhibited the growth and metastasis of orthotopically transplanted triple negative breast cancer cells. Immunofluorescent staining revealed that the adiponectin‐expressing Tregs infiltrated the tumor tissue via a cell‐in‐cell mechanism and were found to be specifically localized around the necrotic areas. Note the merged yellow color, green (adiponectin) and red (FOXP3) in the necrotic area (lower right, without DAPI blue nuclear staining), in the A‐Treg TF attacked triple negative breast cancer.

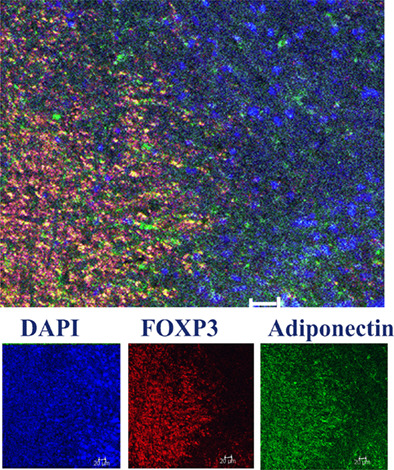

## INTRODUCTION

Triple‐negative breast cancer (TNBC) is characterized by the loss of estrogen receptor, progesterone receptor, and human epidermal growth factor receptor‐2 (HER2). Accordingly, TNBC is resistant to endocrine therapy or HER2 treatment. The aggressive clinicopathological features of TNBC together with its resistance to currently available therapeutics, warrants an urgent need for the development of novel alternate therapeutic options.^1^, ^2^

Adiponectin, a well‐characterized insulin‐sensitizing adipokine, induces autophagic cell death^3^ and apoptosis via fatty acid metabolic reprogramming in breast cancer.^4^ Adiponectin is expressed by a population of T regulatory cells (Tregs) that reside within the complex of thymic nurse cells.^5^, ^6^, ^7^ Adiponectin‐expressing Tregs have promising therapeutic implications in TNBC based on their ability to significantly inhibit mammary carcinogenesis in transgenic mouse.^5^

In our previous study, we successfully expanded a murine T cell fraction containing adiponectin‐expressing Tregs from an experimental thymic tumor model.^7^, ^8^ The T cell fraction together with thymic stromal cells were maintained through coculture. Notably, sorted adiponectin‐expressing Tregs exhibited cell‐in‐cell phenomenon to TNBC cells,^8^ similar to that of the previously reported “HOZOT” cytotoxic Tregs.^9^, ^10^, ^11^, ^12^

The present study aimed to both develop a thymic stroma‐independent T cell fraction with adiponectin‐expressing Tregs and investigate the efficacy of this fraction in suppressing tumor growth in an orthotopic 4 T1 TNBC model.^13^, ^14^

## METHODS

### Cells and culture

The 4 T1‐Luc cells, a luciferase stably expressing mouse mammary tumor cell line, was obtained from the Japanese Collection of Research Biosources Cell Bank (JCRB1447; Osaka, Japan). The detailed characterization of T cells comprising adiponectin‐expressing Tregs has been described previously.^7^, ^8^ Excised tumor cells were composed of two cell mixtures, namely, floating T cells and dish‐attached thymic stromal cells in standard 35‐mm dishes. Floating T cells were cocultured with thymic stromal cells, using a direct cell‐to‐cell contact coculture system.^8^ In the coculture system, thymic stromal cells were gradually decreased until a murine adiponectin‐expressing Treg‐containing T cell fraction (“A‐TregTF”; with the ability to spontaneously expand without the aid of thymic stromal cells), was obtained. Cells were cultured in Dulbecco's modified Eagle's medium (DMEM)‐high glucose (4500 mg/L, Sigma‐Aldrich) with 10% fetal bovine serum.

### Immunofluorescence staining

Immunofluorescence staining was performed according to a previously described procedure.^15^, ^16^ Briefly, cells were incubated with antibodies (1:200 dilution) for 30 min at 4°C. Following incubation, cells were washed with phosphate buffered saline (PBS) and analyzed using a Guava EasyCyte cell analyzer (Guava Technologies, Inc.). The following antibodies were used: rat anti‐mouse CD4 conjugated with phycoerythrin (PE) (clone GK1.5, no. 1102040, Sony) and monoclonal rabbit anti‐mouse CD25 conjugated with fluorescein isothiocyanate (FITC) (catalog no. 50292‐M08H, Sino Biological, Inc.).

The following antibodies were used for fluorescent immunohistochemistry: rabbit anti‐adiponectin (cat. no. GTX107737; GeneTex Inc.) and mouse anti‐FOXP3 (clone 3G3; Proteintech). The tissue sections were washed and incubated with goat anti‐rabbit IgG (H + L) (highly cross‐adsorbed secondary antibody, Alexa Fluor 488, cat. no. A32731TR, Invitrogen) and goat anti‐mouse IgG (H + L) (highly cross‐adsorbed secondary antibody, Alexa Fluor Plus 647, cat. no. A32728TR, Invitrogen). Images were obtained using a confocal laser scanning microscope (Leica TCS SP8) as previously described.^16^

### 4 T1 TNBC orthotopic model

All animal experiments were conducted at Gifu University following the guidelines for animal experimentation and adhering to the Japanese Law for the Humane Treatment and Management of Animals. The experimental protocol was approved by the Animal Care Committee of the Gifu Graduate School of Gifu (approval no. AG‐P‐N‐20220071). Female BALB/c mice used in the study were purchased from Charles River Laboratories, Yokohama, Japan. The mice were housed under 12 h light/dark cycle at 23°C and specific pathogen‐free conditions in isolated and ventilated cages with free access to food and water.

For tumor induction, 4 T1‐Luc cells were inoculated into the mammary fat pads of mice. Subsequently, the mice were injected with adiponectin‐expressing Tregs that were pretreated with 10 μg/mL mitomycin C (Nacalai Tesque) for 2 h. Tumor size was measured using a caliper and the volume was chronologically calculated using the following formula: Volume = width^2^ × length × π/6.

## RESULTS

### Development of A‐TregTF

**FIGURE 1:**
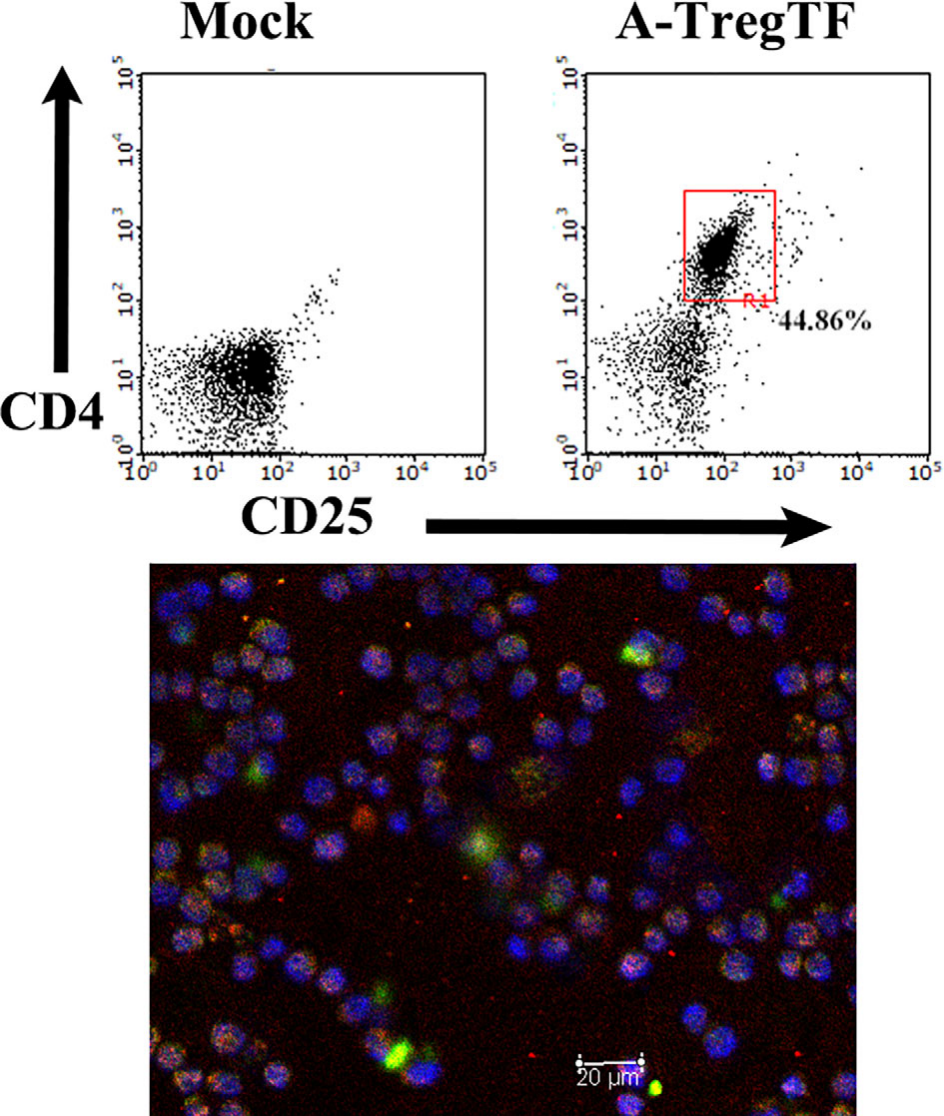
Characterization of cultured T cell fraction composed of adiponectin‐expressing Tregs, A‐TregTF. Upper panel: Control nonstained cells are indicated as mock cells. Representative cell surface staining of A‐TregTF with anti‐CD4 and anti‐CD25 antibodies (A‐TregTF). Lower panel: Cells were subjected to immunocytostaining with specific antibodies. A‐TregTF exhibits immunoreactivity to both FOXP3 and adiponectin. The green signal indicates adiponectin immunoreactivity and magenta represents the merging of red FOXP3 immunoreactivity and blue 4′, 6‐diamidino‐2‐phenylindole (DAPI) staining highlighting the cell nuclei. Scale bar: 20 μm.

The murine tumor model used in this study, from which adiponectin‐expressing Tregs were initially obtained, has been extensively characterized in previous studies.^6^, ^7^, ^8^ In the present study, a spontaneously growing T cell fraction, A‐TregTF, comprising adiponectin‐expressing Tregs was successfully established. The A‐TregTF exhibited surface expression of CD4 and CD25 together with nuclear FOXP3 and cytoplasmic adiponectin expression in T cells (Figure 1).

### A‐TregTF inhibits the growth of orthotopically transplanted 4 T1‐Luc tumor cells in vivo

**FIGURE 2:**
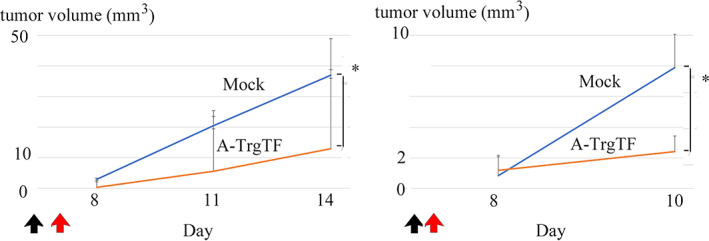
A‐TregTF inhibits the growth of orthotopically transplanted 4 T1‐Luc tumor cells. Female BALB/c mice were implanted with 4 T1‐Luc cells via mammary fat pad inoculation on day 1 (indicated by black arrow). Thereafter, A‐TregTFs were injected at the 4 T1‐Luc transplantation site (day 4 and day 2 in the in the first second experiment, respectively, indicated by red arrow). In both the experiments, A‐TregTFs significantly suppressed the tumor growth of orthotopically transplanted 4 T1‐Luc cells (left; on day 14: right; on day 10, *p* < 0.05, student's *t*‐test).

The inoculation of A‐TregTF into orthotopically transplanted 4 T1‐Luc TNBC mouse model led to a significant reduction in tumor volume. This effect was consistently observed in two independent experiments. In the first experiment, 1.8 × 10^6^ 4 T1‐Luc cells were implanted into the mammary fat pads of female BALB/c mice. Four days later, randomly selected mice were or were not injected with 1 × 10^8^ A‐TregTF at the 4 T1‐Luc transplantation site On day 14, A‐TregTFs significantly suppressed the growth of orthotopically transplanted 4 T1‐Luc cells (*p* < 0.05, student's *t*‐test) (Figure 2, left panel). In the second experiment, we reduced the ratio of target 4 T1‐Luc and effector A‐TregTFs; herein, 6.4 × 10^5^ 4 T1‐Luc cells were implanted into female BALB/c mice.

Two days later, randomly selected mice were or were not injected with 1 × 10^5^ A‐TregTF. On day 10, A‐TregTFs significantly suppressed the tumor growth of orthotopically transplanted 4 T1‐Luc cells (*p* < 0.05, student's *t*‐test) (Figure 2, right panel).

There was no significant development of grossly visible or luciferase‐active metastatic tumors in mice treated with A‐TregTF compared to that of untreated control mice, wherein significant metastasis was detected as reported previously.^14^

**FIGURE 3:**
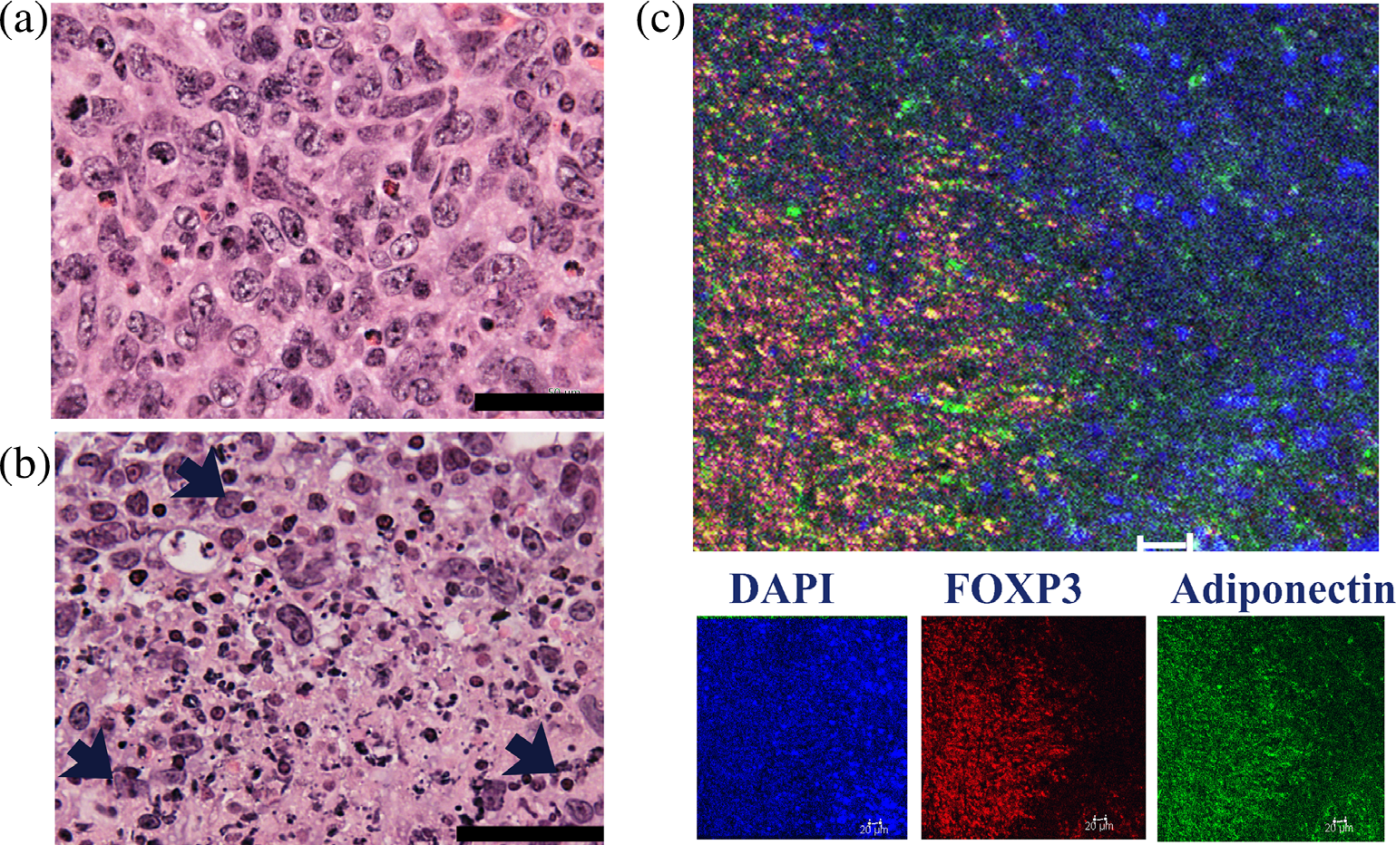
A‐TregTF induces tumor necrosis in orthotopically transplanted 4 T1‐Luc cells. (a) Orthotopically transplanted control 4 T1‐Luc cells exhibit rapid growth in the absence of necrosis. (b) Orthotopically transplanted 4 T1‐Luc cells exhibit dispersed growth with significant necrosis. Note the cell‐in‐cell structure of 4 T1‐Luc cells as indicated by the arrow. Scale bar: 50 μm (a and b). (c) Representative immunofluorescence tissue staining. Merged signals of FOXP3 (red), adiponectin (green), and 4′, 6‐diamidino‐2‐phenylindole (DAPI) (blue) are visualized towards the left side of the necrotic area. Scale bar: 20 μm.

Significant necrosis with dispersed growth was seen in A‐TregTF‐treated‐compared with that in untreated control‐4 T1‐Luc cells, showing robust growth in the absence of necrosis as indicated via histopathological analysis. The representative histopathological findings are summarized in Figure 3.

Notably, several cell‐in‐cell structures were observed around the necrotic areas (Figure 3b). Immunofluorescence histochemical staining revealed infiltration of cancer cells by adiponectin‐expressing FOXP3 positive cells (Figure 3c).

## DISCUSSION

In our previous study, we described a cell‐in‐cell phenomenon observed in sorted adiponectin‐expressing Tregs derived from thymic T cells that were maintained in coculture with thymic stromal cells, with an ability to promote cell death in TNBC cells in vitro.^7^, ^8^

In the current study, we established the efficacy of a T cell fraction consisting of adiponectin‐expressing Tregs, referred to as A‐TregTF, in inhibiting the growth of cancer cells in a 4 T1 TNBC orthotopic model. Histopathological analysis revealed the presence of numerous 4 T1‐Luc cells exhibiting cell‐in‐cell characteristics with adiponectin‐expressing FOXP3 nuclear Tregs localized around the necrotic areas. Together with our previous in vitro finding indicating adiponectin‐expressing Treg as a potential inducer of cell death through cell‐in‐cell phenomenon in TNBC,^8^ the present study proves the potential of A‐TregTF in eliminating TNBC cells via a similar cell‐in‐cell phenomena, both in vivo and in vitro. Furthermore, the results demonstrated that the administration of A‐TregTF into orthotopically transplanted 4 T1‐Luc sites could effectively prevent metastasis.

Previously, a cell‐in‐cell phenomenon was reported to be exhibited by a Treg cell line designated HOZOT in several cancer cells. HOZOT cells are derived from human umbilical cord blood, cocultured with mouse stromal cells, and are characterized as a cytotoxic Treg line exhibiting cell‐in‐cell activity.^9^, ^10^, ^11^, ^12^ The presence of Tregs with cell‐in‐cell activity has also been reported in hepatocellular carcinoma.^17^ Based on these findings, we hypothesize that the A‐TregTF characterized in the present study might be related to HOZOT or other Treg subtypes known to exhibit cell‐in‐cell activity.

In conclusion, our study successfully established a novel T cell fraction consisting of adiponectin‐expressing Tregs capable of spontaneous growth, in vitro, that could effectively inhibit the growth and metastasis of TNBC cells in a 4 T1 TNBC orthotopic model, in vivo. We believe that A‐TregTF holds potential as a therapeutic candidate for regulating tumor growth in patients with TNBC.

## AUTHOR CONTRIBUTIONS

Conceptualization, Chiemi Saigo and Tamotsu Takeuchi; Methodology, Wakana Chikaishi and Toshiya Higashi; Investigation, Wakana Chikaishi, Toshiya Higashi, Hirokatsu Hayashi, Yuki Hanamatsu, and Chiemi Saigo; Formal analysis, Manabu Futamura, Nobuhisa Matsuhashi, and Chiemi Saigo; Resources, Manabu Futamura, Nobuhisa Matsuhashi, and Tamotsu Takeuchi; Writing‐original draft, Chiemi Saigo; Writing – review and editing, Tamotsu Takeuchi; Visualization, Yuki Hanamatsu; Supervision, Tamotsu Takeuchi; Funding acquisition, Tamotsu Takeuchi; Data curation, Chiemi Saigo. All authors read and approved the final manuscript.

## CONFLICT OF INTEREST STATEMENT

No potential conflict of interest was reported by the authors.
